# Dissonances and disconnects: the life and times of community based accountability in the national rural health mission in Tamilnadu, India

**DOI:** 10.1186/s12913-020-4917-0

**Published:** 2020-02-05

**Authors:** Rakhal Gaitonde, Miguel San Sebastian, Anna-Karin Hurtig

**Affiliations:** 1 Achutha Menon Centre for Health Science Studies, Sree Chitra Tirunal Institute of Medical Sciences & Technology, Thiruvananthapuram, Kerala 695 011 India; 2 Department of Epidemiology and Global Health, Umea University, Umeå, Sweden

**Keywords:** Policy implementation, Problematization, Community-based accountability, National Health Mission India, Institutional perspective, Diffusion of innovations, India

## Abstract

**Background:**

There are increasing calls for developing robust processes of community-based accountability as key components of health system strengthening. However, implementation of these processes have shown mixed results over time and geography. The Community Action for Health (CAH) project was introduced as part of India’s National Rural Health Mission (now National Health Mission) to strengthen community-based accountability through community monitoring and planning. In this study we trace the implementation process of this project from its piloting, implementation and abrupt termination in the South Indian state of Tamil Nadu.

**Methods:**

We framed CAH as an innovation introduced into the health system. We use the framework on integration of innovations in complex systems developed by Atun and others. We used qualitative approaches to study the implementation. We conducted interviews among a range of individuals who were directly involved in the implementation, focusing on the policy making organizational level.

**Results:**

We uncover what we have termed “dissonances” and “disconnects” at the state level among individuals with key responsibility of implementation. By dissonances we refer to the diversity of perspective on the concept of community-based accountability and its perceived role. By disconnects we refer to the lack of spaces and processes for “sense-making” in a largely hierarchically functioning system. These constructs we believe contributes significantly to making sense of the initial uptake and the subsequent abrupt termination of the project.

**Conclusions:**

This study contributes to the overall field of policy implementation, especially the phase between the emergence on the policy agenda and its incorporation into the day to day functioning of a system. It focuses on the implementation of contested interventions like community-based accountability, in Low- and Middle-income country settings undergoing transitions in governance. It highlights the importance of “problematization” a dimension not included in most currently popular frameworks to study the uptake and spread of innovations in the health system. It points not only to the importance of diverse perspectives present among individuals at different positions in the organization, but equally importantly the need for spaces and process of collective sense-making to ensure that a contested policy intervention is integrated into a complex system.

## Background

There have been increasing calls for more participatory approaches to governance in frameworks of health system strengthening [1–4]. One of the key components of these frameworks is accountability—described as consisting of the dimensions of “answerability”, the obligation to justify one’s actions, and “enforceability”, the commitment to comply [5]. More recently, calls for accountability have moved from demanding “horizontal” or internal accountability, where the service providers are answerable to their immediate superior in the administrative hierarchy, to more “vertical” or “hybrid” forms of accountability involving communities [6]. There have been a number of initiatives in the past decades that attempted to implement programs to strengthen health system accountability at scale [7, 8]. This is against a backdrop of increasing calls for community participation in the achievement of universal health coverage at the international level [4, 9].

The many calls for increasing community participation in health systems ever since the Alma Ata have been taken up by a number of civil-society led groups (and a few governments) leading to a large body of literature discussing the experiences. However most health systems in post-colonial settings have developed as expert-driven and hierarchical systems. Thus interventions calling for increasing accountability to communities may be considered as innovations that need to be adopted, integrated, and sustained in the various building blocks of these health systems [10].

There is a large body of research regarding the introduction, adoption, and spread of innovations in various systems. The field has moved from the recognition of individual and isolated characteristics of the intervention and the adoption system [11] to recognizing the importance of the “fit” between the innovation and the system, thus bringing to fore the importance of context and history [12]. Further research has shown or highlighted the importance of institutional factors quite apart from the characteristics of the innovation. These institutional factors, referred to by constructs such as “absorptive capacity” or “learning organization” describe a host of characteristics including structural features as well as relations and networks within and between adoption systems [13, 14]. These approaches and frameworks have been applied to the health system too, where a large body of literature has explored the topic from a number of vantage points, including the adoption of clinical evidence, technology, and complex interventions [15–17]. The adoption of innovation studies has pointed to the importance of studying not only the content of innovation, but equally the institutional characteristics of and processes in the system into which the innovation is introduced.

Health systems themselves are increasingly being recognized as complex adaptive systems [18]. This means that the health system is conceptualized as consisting of multiple hierarchical interacting components with complex networks of interaction and influence. Framing health systems as complex systems means that contextual and historical paths of development become key to understanding present functioning as well as understanding the unique response of such systems to the introduction of new programs which may then be considered “innovations”.

In India the National Rural Health Mission (NRHM), introduced in 2005, sought to bring about an architectural correction in India’s health system by undertaking infrastructural development, financing innovations, and governance strengthening. It ushered in an era of increased investment in the public health system and a number of innovations, including those clustered around the concept of “communitization” aimed at increasing the ownership of the health system by the community [19]. While there has been some research around the implementation and impacts [20–22], there is little systematic research on the uptake and integration of these innovations into the health system.

A lot of work has documented the limits of the implementation of processes aimed at strengthening community-based accountability across the world [23, 24]. In the Indian context, research into the implementation of processes attempting to increase community involvement have persistently come up with mixed or limited results [21]. Similarly, recent efforts at the national level in India—under the NRHM—have also met with mixed results, with reports essentially speaking of limited quality of accountability being achieved by the processes [22].

Overall the literature has attributed these mixed results to differentials in power [25], differences in perspective [26], or the co-option of concepts by powerful actors [27]. However, there is little discussion of the institutional processes that lead to the adoption, rejection, or partial uptake of these initiatives, at the level of the health departments in LMIC settings.

In this paper we explore the institutional processes involved in the implementation of community-based accountability of the public health system, in the state of Tamilnadu, India. This initiative, called Community Action for Health (CAH), was introduced in 2008. This research focuses on the institutional processes focusing on the policy making level in the South Indian state of Tamilnadu, through the pilot phase, its subsequent continuation and abrupt termination.

## Methods

### The innovation

The NRHM sought to ensure that everyone has access to essential and quality health care, especially those who were in rural areas. This package of policy recommendations included a number of interventions in key building blocks of the health system such as investment in infrastructure, the setting of Indian Public Health Standards for each level of care, enhancing management capacity, financial flexibility, and what was termed as “communitization” [8]. It can be seen that at least three of these key areas of focus—referred to in the mission documents as the pillars of the NRHM—were related to governance building blocks i.e. standards, management, and community involvement and ownership. One of the key components of communitization was the setting up of a system to enhance community-based accountability.

This component, focusing on community-based accountability, was developed into the CAH project. This included the formation of village health and sanitation committees, their capacity building, and facilitation of their performance of the key tasks of community monitoring and planning. As envisaged in the original project proposal, civil society organizations would be responsible for the creation of village committees, their capacity building, and the facilitation of the processes of community-based monitoring of entitled services at the primary care level. The government would ensure that the front-line staff would be part of the processes where the results of the monitoring were shared and health planning was carried out. Further, it was expected that the government would incorporate these findings into processes leading to the evolution of the states’ annual health plans. The central government funded a pilot project developed by a standing committee of the Ministry of Health and Family Welfare called the Advisory Group on Community Action (AGCA). The AGCA included a number of representatives of civil society organizations who had experience in rights-based approaches to community health and were associated with the *Jan Swasthya Abhiyan* (JSA—the Indian chapter of the People’s Health Movement). This centrally funded pilot project was rolled out in nine states. The idea was that this would enable state governments to familiarize themselves with the process and adopt it after adapting it as necessary. This aimed at increasing the ownership of the process by the state governments. The states were expected to lead the subsequent roll-out and upscaling of the process. As it turned out, the post-pilot phase of the roll-out took very diverse forms. Finally only two of the original nine states in which it was piloted continued implementation of the original model, with the other states either altering it significantly or completely stopping it [28].

### The setting of the research

Tamil Nadu was one of the two states that continued the implementation after the pilot phase broadly following the model originally proposed by the AGCA. The Tamil Nadu state has a three-tier health system (like the rest of India), with health sub-centres (serving 5000 population) and primary health centres (serving 30,000 population) forming the primary care level and managed by a separate directorate dedicated to public health and primary health care services (unlike in the rest of India, where all three levels are managed by one department). Apart from the state-level directorate, each district has a district-level official of the Directorate of Public Health and Preventive Medicine (DPH&PM) who is in charge of the staff and programmes at the primary level. While Tamil Nadu has been widely hailed as a well performing and efficient state [29], studies have also pointed to the centralization of power in the health system [30], which at times has even been characterised as coercive especially when it comes to the achievement of targets [31].

In the case of the CAH project, The State Health Society, which is the unit in charge of funding the various components of the NRHM at the state level, took various steps to enable the smooth implementation and ownership of this intervention by the DPH&PM. These steps included the holding of joint dissemination and planning meetings with various field-level officers of the DPH&PM. At these meetings, apart from the experience of the pilot project, inputs were sought from the front-line workers and their supervisors to adapt the design and evolve an implementation plan that was more relevant to the state. In addition, there was the facilitation of a government order to formalize the commitment of the state, and ensuring that the Director of Public Health was a co-signatory on the MOU for implementation by the civil society organization chosen. Further, the external evaluation of the pilot phase was broadly positive in recommending the continuation of the process [32]. Despite these steps and the presence of supportive officers at both the national and the state level, the implementation continued for only 2 years (2010–2012). After this period, it was abruptly stopped, despite a detailed plan for the next phase of expansion of the project, featuring in the state annual health plan and securing the necessary funding.

The emergence of a space and a government-supported project for the implementation of community-based accountability, its piloting, subsequent roll-out, and abrupt termination against the backdrop of a massive nationwide effort at rejuvenating the public health system forms the focus and the context of this study.

### The conceptual framework

Our research frames the CAH program of the NRHM as a health system innovation that is intended to be introduced and integrated into the public health system with the aim of improving systems of community-based accountability as one component of a proposed “architectural correction” of the health system to be achieved by the NRHM.

**Fig. 1:**
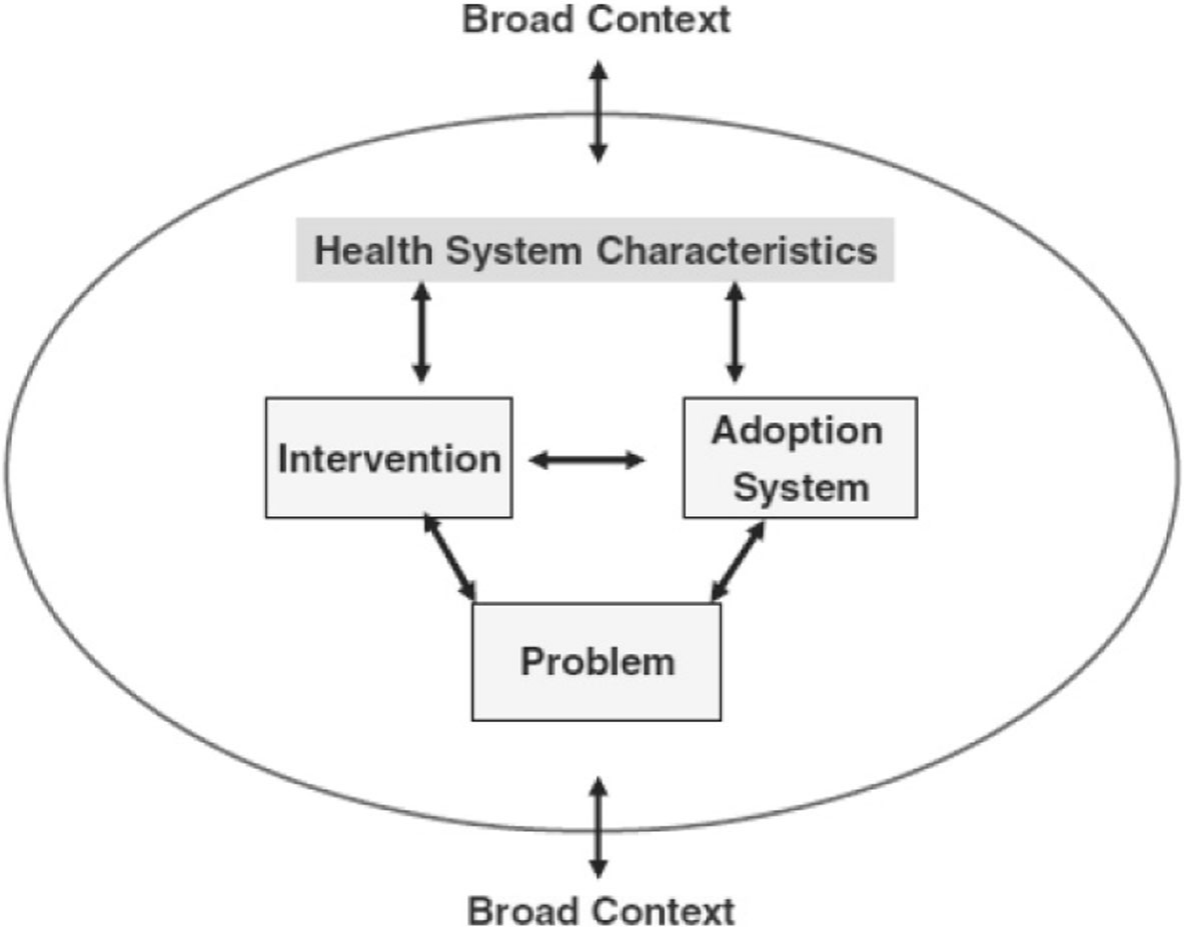
Atun et al.’s framework for the adoption and integration of an innovation into the health care system [10]

The framework suggested by Atun [10] to study the adoption and integration of innovations (Atun’s framework) starts by framing the health system as a complex adaptive system. Further, it focuses on the interactions among five distinct dimensions: the problem, the intervention, the adoption system, the broader health system, and the larger context in which the health system is embedded (Fig. 1).

In the framework the “problem” refers to the social narrative around the issue for which the innovation is being introduced and the sense of urgency surrounding it. In this research the problem which CAH was implemented is access to quality health care as stated in the founding documents of the NHRM [8].With reference to the “innovation”, the framework defines a number of characteristics of the innovation such as relative advantage over other interventions, compatibility (with the system), complexity, trialability (chance to try it out in controlled/observable situations), and observability (possibility to observe the benefits of the innovation). Further, the framework emphasises the issue of complexity, presenting this as three sets of axes, pointing to the importance of the number of times the intervention is to be repeated, by how many stakeholders, and whether the innovation is largely technical or includes behavioural components. The innovation that our research studies is the CAH project, with its components of community-based monitoring and planning, described above.

With regards to the adoption system, the framework points to dimensions such as perception of benefits by different groups, alignment of innovation with values, and legitimacy. In our research the adoption system specifically refers to the State Health Society of Tamil Nadu and the DPH&PM. The broader health system characteristics refer to the overall alignment in terms of regulatory and accountability frameworks, and integration with already existing management and financing mechanisms. This broader health system is of course that which is found in the state of Tamilnadu. Finally, the dimension of the broader context refers to the interplay of the demographic, economic, political, legal, ecological, socio-cultural (including historical legacies), and technological factors in the environment in which the foregoing considerations (the problem, intervention, health system characteristics, and the adoption system) are embedded. This refers in our research to the overall macroeconomic and political situation in Tamil Nadu specifically, but also in the whole country.

In this research we have used the framework to guide our exploration of the implementation process. We have applied the various dimensions and particular aspects of each to guide our analysis of the material collected during interviews with key actors in the implementation process as described below.

### The study design

We adopted a qualitative study design, using in-depth interviews to document and make sense of the processes that took place during the adoption, implementation, and subsequent abrupt discontinuation of the CAH project in the state of Tamil Nadu. The focus of our research was the state level, rather than at the district and community levels as it is the decisions at this level that constrain the way the policy finally rolls out at lower levels.

### Selection of research participants

The research participants were officials and civil society leaders at the national and state level who had played an active role in various stages of the implementation of the CAH project since the time it was designed at the national level and piloted in the state in 2008 until its discontinuation in 2012. The nature of the implementation in the Department of Health means that in the initial stages of implementation (before an intervention is fully upscaled and integrated), it is the responsibility of only one or at the most two junior officers at the state level, who report to the senior policy makers. Thus, those who were involved in the actual implementation of the CAH project, and who could provide insights were very small in number. In all we interviewed nine individuals (with some interviewed on more than one occasion). Of these, five were government officials (one at the national level and four at the state level), and the rest were representatives of civil society.

### Data collection

The first author developed a frame of key individuals at different positions in the health department who were directly involved in the implementation of the CAH project. Individuals from every level of the state level hierarchy were included in the final list of interviewees. The first author (RG) also developed the overall interview guide, which was finalized through discussion with the other authors. He approached each of those identified and conducted the interviews at locations and times most convenient to the participants. The interviews were conducted in English and lasted 1 h on average. The interviews focused on the participants’ understanding of the concept of accountability as well as her/his understanding/perspective and insights on the implementation of the CAH process. Interviews were then transcribed and the transcription checked against the original recording by RG. Once finalized, both the recordings and the transcripts were stored as secure soft copies. A copy of the interview guide used can be found as supplementary material file (Additonal file 1).

### Data analysis

In this study we analysed the data thematically [33, 34], coding into the various dimensions of the Atun framework [10] and further trying to understand the sub-themes under each of these. After familiarizing himself with the data, RG proceeded to do an initial coding that remained close to the data. Further steps included arrangement of these codes into the various dimensions of the framework and discerning the emerging narrative with reference to the actual implementation process. At each step the evolving analysis was discussed among all the authors.

## Results

The CAH project was an innovation to strengthen community-based accountability that was introduced by the NRHM. States governments were expected to roll out the process after an initial pilot project. In this section we arrange our findings using the various dimensions of Atun’s framework (Fig. 1).

### The innovation

The processes of the CAH project required multiple repetitive activities like monthly village committee meetings, regular training, and six-monthly monitoring and planning activities. It also involved a large number of stakeholders, including village committee members, local government representatives, NGO staff, and front-line health workers. Moreover, it involved a very complex behavioural component including attitudinal change among both community and front-line health workers with regards to accountability. Thus, using the three axes suggested by the framework to assess complexity, this may be described as a highly complex intervention—which substantially increased the challenges of its integration.

With respect to the perceived advantages of the intervention, what emerged from the interviews were two distinct patterns of responses. One group of actors saw the intervention as a logical step towards health system strengthening. They recognized its clear advantages over the way the department had functioned in the past.

*It was something that appealed . . . inherently. . . that is instead of external monitoring of anything if we could actually get the community to work in and around the health system, I felt that the synergies could be very very spectacular*. (IDI 1—government official)

Another group of actors, on the other hand, described it as an irritant, which against the grain of the health system.

*But the feedback I got . . . is, people are stamping on my feet; I pretend not to cry. Because some officer is supporting. That is the view. . .. That’s it. So, they were not comfortable with the programme*. (IDI 2—government official)

Various steps were taken to ensure that the public health staff could familiarize themselves with the CAH project. This included the implementation of a pilot project, a state-level dissemination meeting, and commissioning an external evaluation at the end of the pilot phase. These have been detailed in the section above, “The setting of the research”. These were systematic attempts at ensuring that officials and front-line providers have a chance to try it, and give their feedback and suggestions for adaptation before adoption. These steps took care to enhance the “trialability” dimension of the framework. Further, the state-level dissemination meeting gave a space for those officials who were part of the pilot process to share their experiences with other officials, thus enhancing in addition the “observability” dimension.

### The adoption system

According to the framework, the main components in the adoption system that are relevant for the adoption of an innovation are the alignment of the intervention with various components of the system and its perceived legitimacy within the adoption unit. By the adoption system we are referring here to the State Health Society and the Department of Public Health and Preventive Medicine which was the primary implementing department under the Department of Health in the state. Most of the interviewees spoke of the fact that while the CAH project had been implemented in the state with the support of senior officials of the government, it failed to develop a buy in from the other officials in the department (even at the state level). A few of the key points that emerged were.

There seemed to be a fundamental resistance among some with a process that organizes communities to monitor.

*“The monitoring component included (in the past) … petition writing, complaint writing … holding grievance days. But when an organized monitoring came into existence, there was some kind of opposition from the body (sic).*” (IDI 3- Government official)

While at the national level there was a clear mandate for the involvement of NGOs in the initial phases of implementation, at the state level this support was complicated. While some bureaucrats mentioned the need for a trusted partner with whom they could push the innovation forward due to flexibility in their functioning and their grass root presence, others felt that NGOs were irritants who were disrupting a system of relationships built up over years.

One of those interviewed noted that in general the project was not able to generate any buy-in internally. Similarly another pointed out that, it was clear that the project was not understood and accepted within the department despite support from the highest level.

This builds on a basic mistrust of NGOs that characterises the system. Historically when recalling various instances of community monitoring those interviewed pointed to instances when community donated land for buildings, or participated enthusiastically in training programs etc., Any feedback from the community was described as being individual and on a one on one basis with the concerned doctor / person in charge. This was the community participation the department was used to. Thus, when the CAH project, which included the regular monitoring of the health care services by a committee with a checklist etc., it was seen as disturbing the status – quo. Describing the doctors reaction to NGOs leading the program one of the officials said,

*even the person who used to come and say about it (complained) started going somewhere else (reporting to the NGO facilitated process). That is the real problem which the doctors have faced*. (IDI 3 – government official)

### The health system

Those interviewed noted that while the idea of community-based accountability was good in theory, it went against the grain of the culture of the health system as it had developed in Tamilnadu. Thus respondents pointed to the largely utilitarian nature of the government–community relationship in the state, in the past.

*They are using the community for, I think, programme needs or personal needs or political needs or whatever it is*. (IDI 3—government official)

Given this sort of a culture of functioning, it was noted that,

*We were never able to put it in place . . . because . . . the difficulty of getting in external monitoring systems which are more connected to the ground reality is a real problem in government*. [IDI I—government official]

One of the key aspects highlighted by the respondents was that the form of governance within the health system was as they termed “personality based”. This in the light of the earlier comments on the hierarchical nature of the system meant that there would be little scope for a process of collective sense making. It was as if the system would mould itself to what the person on the top liked or wanted.

*And the system actually moulds itself to the top. It is the beauty of it... in Tamilnadu, you think this guy is like this, the system won’t accept him. No. Actually what will happen is, the entire system will accept him for a particular period. Somehow it moulds itself. That’s the beauty of Tamilnadu*. (IDI 3—government official)

Thus there emerged a situation in which most planning was focusing on extremely short-term and output-focused goals, with little long-term and strategic-planning vision.

*Everything is short-term goals. So today I do something, tomorrow people should see that . . . so nobody has long-term goals. . . . For short-term goals they do whatever they want*. (IDI 3—government official)

Further, participants pointed out increasing political interference in the autonomous functioning of the directorate. The allocation of infrastructure is an example; while earlier it would be based on a measure of population need, increasingly allotment was based on political pressure.

*The MLA (elected representative) or the government says that, you choose this area for a primary health centre or sub-centre, we have no say over it. But for the community, we know that, that is not a proper place*. (IDI 4—government official)

Similarly, the respondents also alluded to increasing financial constraints and interference in policymaking from bureaucrats as constraining the autonomy of the department. It was reported that this started since the decade of the 1990s.

Those interviewed also pointed to a stagnation of services that seems to have occurred over the last two decades. Thus many of the community-level training programs that used to take place were stopped, and a number of services that used to be provided at the village level were now only at the institutional level. One service which was affected was the postnatal service. Thus, while respondents noted the drop in postnatal care as an exemplar of weakening community services, it was pointed out that when deliveries were being conducted in the health sub-centres (5000 population level) by the village health nurse herself (a practice that was continued until the early 2000s), postnatal care was high, as there was obvious motivation and interest in following up with the person she had delivered. However, with policy change and shift of deliveries to the primary health centres, the front-line workers lost their motivation, leading to the erratic service delivery quality.

*Now there is nothing. They are not bothered at all. . . .You have lost the confidence of the worker. . . . Some process, confidence, and faith was there. That faith was eroded like anything*. (IDI 4—government official)

Further, given the fact that the process entailed the identification of gaps in entitlements—many of the causes of which were systemic—there was a sense of tension, in the sense of not being able to respond to the findings of the monitoring in any meaningful way. This along with the fact that the system was hierarchical meant that the front-line workers could not see the monitoring process as the first step in co-production, but could only see the whole process as one of evaluation and failing, belying the expectations of the senior policymakers who were attempting to introduce this innovation.

### The broad context

While discussing the issues pertaining to the broad context, several observations were made by the interviewees.

One was about the general reticence of the India bureaucracy to give up or share power. This was pointed out both at the national and the state level. While there were constitutional mandates for power sharing, bureaucrats in general were perceived to be wary of sharing powers with levels of government below them.

*But the power structure being as it is in our federal system, that the federal government is very chary of giving powers to the states, and the states are also chary in giving powers to the panchayati raj (local self government) despite, you know, our constitution*. (IDI 5—government official).

While those who made this comment made it in reference to the *Panchayat Raj* Act, by which power sharing with local self-government bodies was mandated, it was meant to highlight the fact that processes such as CAH which were aiming to establish community-based processes with a redistribution of power were unlikely to succeed.

Few respondents referred to the way in which the state government stifled the expansion of the adult literacy campaign (*Arivoli Iyakkam*) when it was perceived to be taking on a “political” nature—or as put by one of the respondents, when they felt that they were no longer in control and communities were becoming more and more empowered.

*Changes started happening very rapidly . . . this was particularly aggressive among women and youngsters . . . so one of the major questions that arose in the mind of the high-level decision makers was how to take the process forward. . . . Will a huge aggressive movement be created? Will women get together and start a struggle? . . . They started posing a number of financial and administrative questions . . . which created necessary data . . . but was also useful in suppressing the movement*. [IDI 6—NGO representative]

These responses point to the system-level resistance to power sharing in reality, despite rhetoric about architectural correction and the commitment to communitization.

Finally, a point was also made about the fact that as communities got more “developed” they became less and less dependent on government services in general and public health services in particular, as they would prefer private services. The public sector weakened by neglect and constraints in funding was left increasingly for the poor and marginalized. In such a situation, with the influential middle class moving away from public-sector services, it was pointed out that there was no motivation either for people to hold the government accountable or indeed for the government to expect people to question it with regards to entitled services. Thus, there was a general sense of indifference which formed the overall backdrop of any such interventions being introduced.

### The problem

It is clear from the above sections that there is divergence in the way different groups of officers viewed the intervention. What becomes clear from the interviews is that this divergence in perspective extends from the way the problem was framed in the first place.

The NRHM documents clearly identify access to essential and quality health care services, especially to those living in rural areas, as the issue in need of solving. The CAH process was recognized by all actors as part of a range of interventions under the “communitization” dimension of the NRHM. While the focus was clear enough, the range of suggested innovations (solutions) to overcome the problem included increasing investment and infrastructure, defining standards at each level of the public health system, deploying community health workers, and increasing the flexibility of financing and capacity to manage programs at various levels of government. This particular range of solutions chosen seems to present the lack of access as being due to gaps in infrastructure and efficiency of the system, which could consequently be solved by enabling the community to hold the system accountable for the provision of entitled services, as well as increased community involvement in health planning (through the CAH project). This was contested by the civil society representatives who were part of the AGCA. They instead framed the problem as being essentially one due to a power differential between the community and an essentially expert driven health system. They saw the CAH as a way of engaging with and reducing this power differential.

However research in Tamil Nadu revealed that there were in fact two competing perspectives within the health department itself. The framing described above which was emergent at the national level was subscribed to by one group of officers in the state. As described above, they agreed that training communities about entitled services and enabling them to hold the front-line workers accountable, with active input from NGOs, would improve the situation. However, research reveals another group of actors who framed the problem of ineffective coverage of the community by services due to increasing interference by the political class and increasing constraints in funding (since the 1990s). For this group the solution lay in less interference from the political class and more funds to the public health system. Thus while there is a difference in the way the national government and the civil society groups framed the problem and hence the solution, research also reveals the presence of two distinct framings of the problem within the health department in Tamilnadu.

#### The path of the CAH project in Tamilnadu

##### Origins

It was pointed out by most of the interviewees that many of the individuals from within the government who played a key role in the conceptualization, piloting, and rolling out of the process, both at the national and state level, actually had multiple exposures to forums and ideas outside formal government. These officers had served in international developmental organizations, had been involved in doctoral studies in multidisciplinary academic units, and had been exposed to and influenced by interactions with a number of leading civil society organizations, especially in the field of accountability and governance.

*X1 came to this position immediately after his lien in XXX . . . and he came full of ideas . . . decentralization, participation, governance. Similarly, X2 was in an NGO many years ago . . . so doing something he had done, he came in with a spark in the eye*. [IDI 5—national-level NGO leader]

Most participants considered the officers who were at the helm during the initiation of the program as boundary spanners. They were charismatic and well respected.

*XX’s (referring to senior officer) advantage was that s/he (for anonymization) too understood the issue the way I did. . . . I think XX was spectacular . . . so could see that I had caught on to something*. [IDI 1—government official]

However, after the pilot phase, the responsibility for implementation moved to the state directorate of public health, and in time once the officers who initiated the program shifted as part the routine shuffling of officers in the Indian bureaucracy, the onus of sustaining the implementation was on a different set of officers. However, the new officers at the state level who were in the lead of the implementation were described as:

*Basically the people who have come up right now have very little exposure to other systems and everything . . . its like a horse’s view. Still they think that our system is the best system and we’ll ride with our system*. [IDI 2—government official]

The officers who introduced the program and supported it initially, also spoke of support from within the department from their senior officers, and equally importantly of the “trusting” relationship they shared with the civil society organization which was implementing the project. However, within the department it was obvious that not only was there a group of officials who had a different understanding and framing of the issue, but that these officials did not express their dissent due to the hierarchy within the system and the expressed view of the officer in the apex position.

*“However the community monitoring component was included because of a certain person (naming an official) … to a limited level only … .so we have limited it as far as the Tamil Nadu state is concerned”.* (IDI 3 – government official)

This is made even more clear by the fact that the moment the senior official who was supporting the project moved, the group of officers who did not support the framing of the project as either necessary or legitimate were able to abruptly stop its implementation.

## Discussion

The CAH project was an innovative attempt at strengthening community-based accountability, introduced against the backdrop of the NRHM, a massive program to strengthen the public health system. It was premised on active involvement of civil society organizations and increasing the accountability of the public health system to the communities they served.

The CAH was a complex community-based intervention. As described above and in more detail elsewhere [28], despite finding a prominent place on the policy agenda, many attempts at facilitating its implementation, positive evaluations, and very positive political and bureaucratic support, the program was abruptly stopped. This research focused on official and civil society representatives in the state of Tamilnadu who were directly involved in its implementation, to study the roll-out of this project. Framing it as an innovation, the study has looked at the diffusion and integration of this innovation in the state health system. What emerges is what we have termed as “dissonances” and “disconnects” within the key organization meant to adopt and implement this innovation. This we argue set the stage for its termination despite intense efforts to facilitate its implementation and integration.

By “dissonances” we are referring to the diverse perspectives that were documented with regard to the particular innovation in question. The significance of this is the existence of divergent perspectives over the way issues were framed within the same adoption unit, the DPH&PM, and the State Health Society of Tamil Nadu. By “disconnects” we refer to the lack of spaces, processes, and an environment that supports what has been described in the literature as “collective sense making” [16, 35, 36]. This is especially important in the presence of divergent perspectives (dissonances) in the same department. In the following subsection we draw on the results presented above and through the constructs of “dissonances” and “disconnects” place them in the context of the larger literature of diffusion of innovations.

### Dissonances

To us the diverse perspectives on the innovation represent underlying differences in the way issues were problematized. A more detailed engagement with these underlying perspectives and belief structures can be found in [37]. The underlying problematization of the issues addressed by the innovation is a critical but neglected dimension in most popular frameworks that discuss the adoption and integration of innovations into health systems [10, 12, 38]. In the research reported here they emerge as a key determinant of the way in which different groups of officials within the adopting system perceive and consequently assess the innovation.

The key finding of the research was the identification two distinct groups, holding divergent perspectives, within the department of health. This has been further confirmed and elaborated in earlier work [37]. The references to increasing financial constraints and the erosion of autonomy of the technical officers by the bureaucrats referred to earlier point to the possibility of these representing the persistence of past governance regimes—as the state transitions from one driven by a welfare philosophy to one increasingly driven by a neo-liberal philosophy [39] or managerialism [40]. This sort of persistence has been described in the literature on institutional change and has been termed as partial de-institutionalization [41].

This difference in perspective has direct implications for how a particular innovation is assessed. This underlines that innovations rarely have isolatable or permanent characteristics. On the contrary, characteristics emerge in specific contexts. This move towards dynamicity is reflected in the literature by the calls to focus on innovation–system fit rather than stand-alone characteristics of innovations [12].

We have shown that in the case of a complex social innovation like CAH, perceived relative advantage of the innovation, and the conflict over this, is more important than trialability and observability for its sustained adoption and integration. Importantly this research points out that the conflict is at a much deeper level – that of meaning. This research points to differences at the level of problematization. Thus the divergence in the dimensions of perceived advantage is linked to divergent deeper beliefs structures within the department of health. Given this deeper level of conflict, the attempts by officers to increase the trialability and observability, through the pilot project implementation, external evaluation, dissemination meetings, and joint processes to move the intervention post the pilot phase (described above in “The setting of the research”) did not seem enough to ensure adoption and integration into the system.

We believe that the persistence of rival problematizations points to the dynamic and iterative processes of changes that are taking place currently in the various public-sector administrative apparatuses, especially in developing country health systems. It has been pointed out in the literature that both the participatory as well as the neo-liberal projects share core notions such as citizenship, participation and civil society, but with very different meanings [27] and therefore with different implications for the form a particular aspect will take during implementation. It is being argued here that despite its origins in the participatory framework, the idea of monitoring may have a much more restricted meaning when implemented in the context of neo-liberal informed governance. Further, the present consensus and dominant perspective may not be stable and could be displaced at times when the balance of power is not in its favour. This was what probably happened when a few years into the implementation of the CAH project the key champions both at the national and the state level were shifted, and a range of new officers took over in both levels. As noted in the literature, “organizational realities also contain marginal, submerged or more openly competing counter or alternative institutions that are both within and without the organization. In ‘unsettled periods’, in times of crisis and change, or when competing institutions offer alternatives, institutional symbols and practices may turn from second nature into ‘resources manipulated by individuals, groups and organizations” [41]. This was reflected by the fact that the balance of perspectives changed when key officials at the national and state level were shifted, underlining the inherent instability in such balances.

### Disconnects

The other key emergent construct is that of “disconnects”. We refer here to the lack of spaces and processes for collective meaning making. Thus, while there were clearly differences in the way different officers (or groups of officers) problematized the issue, given the hierarchy inherent in the department there was the lack of space (s) to engage with these deep-seated differences. This is especially important in hierarchical systems where dissent is avoided. What seemed the key determinant of the continuation of the project was the presence of individuals in key positions of power and their ability/commitment to a particular idea/innovation. Given the overall lack of consensus / common understanding of the project (especially at the deeper meaning level) that was developed (or indeed that had a chance to develop given the lack of spaces), the moment the officer in power shifts the continuation of that project becomes unstable.

It was clear that new ideas and innovations were introduced and fostered by leaders who may be characterised as boundary spanners [12], who had wide experience of interacting with a number of agencies outside government. The lack of spaces for discussion and debate over these new ideas, especially from the perspective of those from the field, meant that the integration of these innovations into the day-to-day functioning of the health system was at best unstable.

These findings further relate to a construct that has been discussed in the literature on adoption of innovations, called “absorptive capacity” [14, 42]. One of the key aspects of this construct is the number of processes that are undertaken to incorporate the newly introduced innovation into the already existing knowledge. It is these active processes that enable or fail to enable recommendations based on research findings to be converted into something relevant for the local situation. These processes require time, space for discussion, and an environment where diverse groups of individuals at different levels of the health system have a chance to express and share their ideas, priorities, and misgivings. This is especially evident as it does not only seem to be a problem about a disagreement with regards to the interpretation of research, but seems to be a much deeper-level divergence in the way the issue is problematized. Thus, while the system may work efficiently in terms of outputs, there is a sense of internal disconnects in terms of collective sense making and organizational learning, probably having a major impact on the absorptive capacity for such complex innovations.

This paper speaks to the complex and not very well studied phases of policy process that follow emergence on to the policy agenda. This phase been described as “the bit in the middle” in a paper which draws on experiences described in 28 research papers from across the world and identifies seven distinct activities during these phases “generation of policy alternatives, deliberation and/or consultation, advocacy of specific policy alternatives, lobbying for specific alternatives, negotiation of policy decisions, drafting or enacting policy and guidance/influence on implementation development [43]. Other key aspects of this paper that have been described in the literature, include the changing governance regimes in Low and Middle Income Countries and the impact it has on accountability [44, 45], and the influence of the neo-liberal agenda on the way community participation is implemented in Latin America [27]. Our paper thus is relevant and adds to this body of literature that engages with the process of policy implementation especially of contested concepts like community participation, in settings that are transitioning between different regimes of governance.

### Limitations

This research focused on a particular policy regarding community-based accountability, and focused only on the apex administrative level in the state. The policy studied is contested in nature. Thus, the research does not purport to explain the implementation of all policies at all levels, but hopes to provide in-depth understanding of the processes and mechanisms at one level, as a contribution to understanding the larger picture. The analytical approach taken in this research is only one of many possible. However, given the data, the involvement of the first author in the actual implementation of this project, and the balance provided by having multiple authors (each with experience in a wide range of settings), we believe that this perspective is an important one that gives particular insights into the implementation process. While the research was set in the somewhat unique circumstances of the state of Tamil Nadu, we believe that the level of abstraction we have achieved with the analysis would enable the key constructs to provide valuable insights to analysing implementation in diverse locations.

## Conclusions

This study contributes to the overall field of policy implementation, especially the implementation of contested interventions like community-based accountability. It highlights the importance of “problematization” as dimension not included in most currently popular frameworks to study the uptake and spread of innovations in the health system. It points not only to the importance of diverse perspectives present among individuals at different positions in the organization, but equally importantly the need for spaces and process of collective sense-making to ensure that a contested policy intervention is integrated into a complex system. Given the emergence of divergent ways of understanding a situation, it follows that the same intervention might be assessed differently by different groups of actors in the same setting depending on their underlying problematization. This points to the importance of assessing the innovation–system fit (which is a dynamic construct) rather than the static and stand-alone characteristics of an innovation. Further, in situations where multiple perspectives arise, the perspective that dominates depends on the various institutional resources that particular groups can deploy.

Secondly, we have pointed out the importance of having spaces or the institutional environment to enable these deeper differences to be worked on and sorted out. We show that especially in hierarchical systems, where these spaces and the environment for discussion and debate do not exist or are limited, implementation depends more on individuals in key positions of power (and their particular problematizations) rather than on an institutional-level consensus and understanding.

This situation of dissonances, especially in postcolonial and transitional settings, in which there is the likely persistence of multiple institutional governance regimes with states transitioning from welfare to neo-liberal regimes, makes it essential to have the presence of spaces, processes, and environments to discuss and debate these deeply divergent perspectives. In the absence of such spaces, i.e., in the context of disconnects, the integration of innovation into health systems is likely to remain unstable. What also emerges from our research is that processes of adoption and integration of innovations in health systems are not one-time processes that depend on any inherent or stable characteristics of either the innovation or the adoption unit (embedded as it is in the larger health system, which in turn is embedded in the macro context). The implementation and integration of complex interventions, like the CAH in Tamil Nadu, represent a dynamic process. This brings to the fore the fact that implementation of and the subsequent integration of such complex interventions requires constant effort and cannot be taken for granted, even if present on policy agendas. This is especially so in the case of such innovations that entail fundamental changes at the level of problematization of issues.

## Supplementary information

**Additional file 1.** Interview guide.

## Data Availability

Given that this is qualitative interview and that we have assured the interviewees of confidentiality, especially given the sensitive nature of the material - it will be available only on request, under special circumstances after clearing with the ethics committee / authors group.

## Abbreviations

AGCA: Advisory Group on Community Action
CAH: Community Action for Health
DPH&PM: Directorate of Public Health and Preventive Medicine
JSA: *Jan Swasthya Abhiyan* (the Indian Chapter of the People’s Health Movement)
MOU: Memorandum of Understanding
NRHM: National Rural Health Mission
