# Supplementary material for: Dissonances and disconnects: the life and times of community based accountability in the national rural health mission in Tamilnadu, India
Source: BMC Health Serv Res. 2020 Feb 5;20:89. doi: 10.1186/s12913-020-4917-0 (PMC7003366; doi:10.1186/s12913-020-4917-0)
Supplement: Supplementary file 1 — Additional file 1. Interview guide. [file 12913_2020_4917_MOESM1_ESM.docx]

Community Based Accountability in Tamil Nadu

Interview guide

- How would you describe an accountable health system? How would this look like? What features would it have? What is the role of communities in such systems?
- Have you ever been directly involved in or heard off any programs that invoked community participation and accountability? What was your experience? Your general feelings about such programs. (This question does not refer to CAH related experiences).
- You have been directly involved in the implementation of CAH - Describe the program for me? what were some of the aspects of the program that you particularly liked? Why?
- What were some of the aspects of CAH that you would have like to see implemented more vigorously? What were the reasons in your opinion for not being able to implement these?
- What are some of the important roles that communities, NGOs and the government has to play in the implementation of CAH – highlight with examples where ever possible.
